# P-2111. Heart Transplant Candidates and Strongyloides stercoralis: Screening Outcomes and Testing Rates in Solid Organ Transplants

**DOI:** 10.1093/ofid/ofaf695.2275

**Published:** 2026-01-11

**Authors:** Katia Ashy, Drew W Charles, Alexandra G Mills, Scott R Curry, Ruth O Adekunle, Yosra Alkabab, Rachel Burgoon, Eric G Meissner, Divisha Sharma, Delaney Smeak, Ryan J Tedford, Courtney E Harris

**Affiliations:** Medical University of South Carolina, Charleston, SC; Medical University of South Carolina, Charleston, SC; Medical University of South Carolina, Charleston, SC; Medical University of South Carolina, Charleston, SC; Medical University of South Carolina, Charleston, SC; MUSC, Charleston, South Carolina; Medical University of South Carolina, Charleston, SC; Medical University of South Carolina, Charleston, SC; Medical University of South Carolina, Charleston, SC; Medical University of South Carolina, Charleston, SC; Medical University of South Carolina, Charleston, SC; Medical University of South Carolina, Charleston, SC

## Abstract

**Background:**

*Strongyloides stercoralis* is a soil-transmitted roundworm that can cause human infection with high rates of mortality and morbidity. Traditionally thought to be found in subtropical regions, increasing incidence show endemicity in the Southeastern United States and Appalachia. In 2019, the Medical University of South Carolina (MUSC) implemented universal serologic screening to identify prior *Strongyloides* exposure during evaluation for heart transplant candidacy. Despite the American Society of Transplantation’s recommendation for serologic testing in all solid organ transplant (SOT) candidates who reside in or have extended travel to endemic areas, limited data exists on the incidence of *Strongyloides* in the pre-transplant population. Our study aims to determine the incidence of prior *Strongyloides* infection among all SOT recipients at MUSC.

**Table 1.**
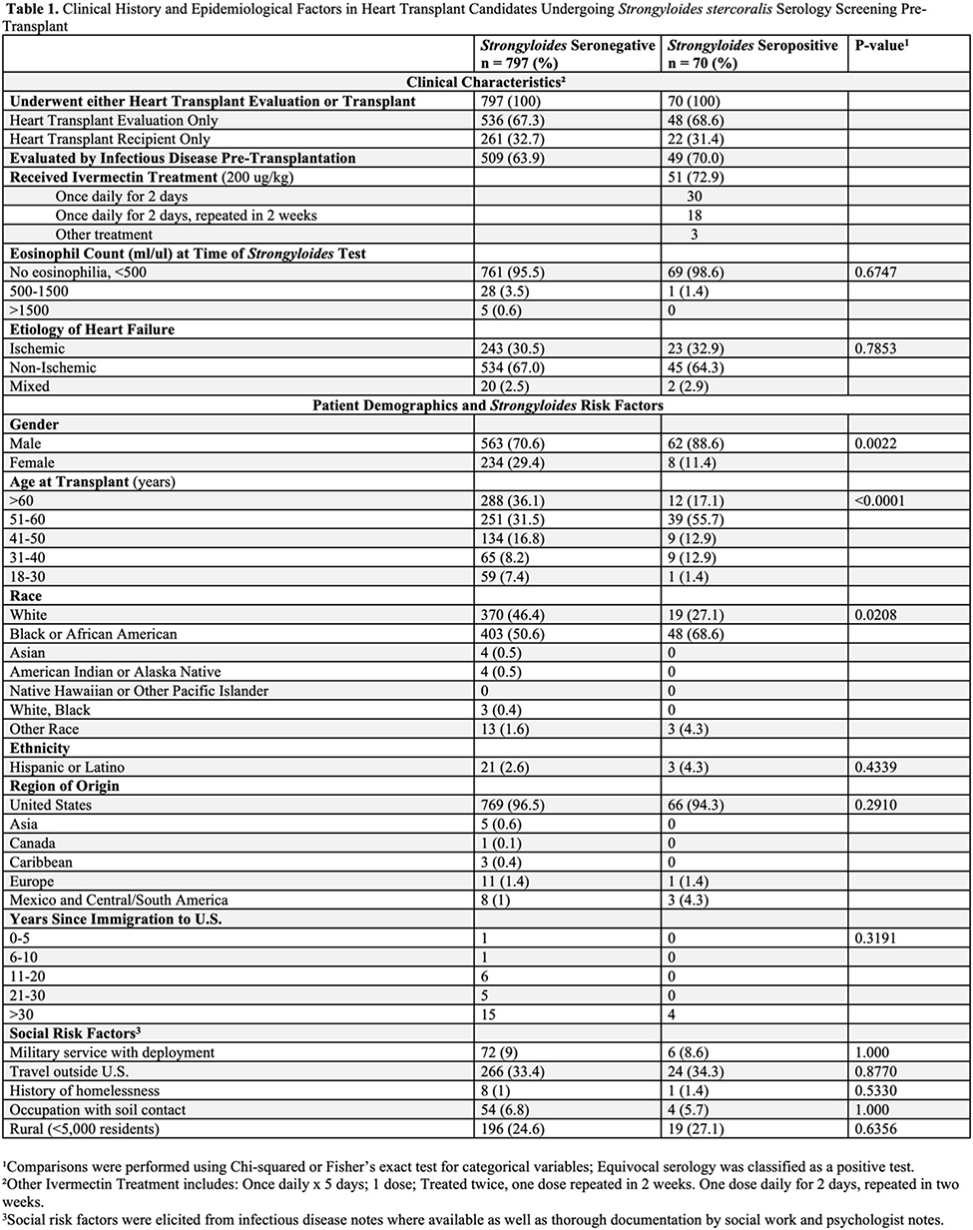
Clinical History and Epidemiological Factors in Heart Transplant Candidates Undergoing Strongyloides stercoralis Serology Screening Pre-Transplant

**Table 2.**
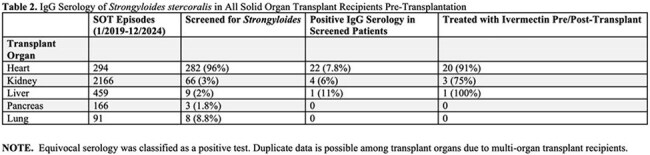
IgG Serology of Strongyloides stercoralis in All Solid Organ Transplant Recipients Pre-Transplantation

**Methods:**

A retrospective chart review was performed of all heart transplant candidates and recipients evaluated at our center between January 1, 2019-December 31, 2024, with data extracted from the electronic medical record (EPIC). Data included patients’ demographics and epidemiological risks for exposure at time of serologic screening. Data on all SOT *Strongyloides* testing and treatment was collected through the Slicer Dicer tool in EPIC.

**Results:**

A total of 867/885 (98%) patients who underwent heart transplant evaluation were screened for *Strongyloides* and 70/867 (8.1%) were IgG seropositive. Patients who were male or African American had higher relative rates of *Strongyloides* seropositivity. No traditional social history exposures, including occupational soil exposure or travel outside the U.S., differed between groups. 20/22 (91%) seropositive heart transplant recipients received ivermectin, and no cases of disseminated *Strongyloides* were recorded in our cohort. *Strongyloides* screening for all SOTs was low compared to the universal screening in heart transplant candidates.

**Conclusion:**

Seropositivity rates differed based on age at time of transplant, gender, and race. As social history questions proved unreliable to predict for *Strongyloides* seropositivity, our results reaffirm the recommendation for universal screening in heart transplant candidates, which we have extended to all SOTs at our organization.

**Disclosures:**

Rachel Burgoon, Pharm.D., Merck: Grant/Research Support Ryan J. Tedford, MD, Abbott: Advisor/Consultant|Abbott: Honoraria|Abiomed: Board Member|Acceleron/Merck: Advisor/Consultant|Acceleron/Merck: Honoraria|Acorai: Advisor/Consultant|Acorai: Honoraria|Adona: Advisor/Consultant|Adona: Honoraria|Alleviant: Advisor/Consultant|Alleviant: Honoraria|Aria CV Inc.: Advisor/Consultant|Aria CV Inc.: Honoraria|Boston Scientific: Advisor/Consultant|Boston Scientific: Honoraria|Cytokinetics: Advisor/Consultant|Cytokinetics: Honoraria|Edwards LifeSciences: Advisor/Consultant|Edwards LifeSciences: Honoraria|Endotronix: Advisor/Consultant|Endotronix: Honoraria|Gradient: Advisor/Consultant|Medtronic: Advisor/Consultant|Medtronic: Honoraria|Merck: Advisor/Consultant|Merck: Honoraria|Morphic Therapeutics: Advisor/Consultant|Morphic Therapeutics: Honoraria|Pulmovant: Advisor/Consultant|Pulmovant: Honoraria|Restore Medical: Advisor/Consultant|Restore Medical: Honoraria|Tempus AI: Advisor/Consultant|Tempus AI: Honoraria|United Therapeutics: Advisor/Consultant|United Therapeutics: Honoraria

